# Intelligent Fault-Diagnosis System for Acoustic Logging Tool Based on Multi-Technology Fusion

**DOI:** 10.3390/s19153273

**Published:** 2019-07-25

**Authors:** Xiaolong Hao, Xiaodong Ju, Junqiang Lu, Baiyong Men, Jing Zhou

**Affiliations:** 1 Downhole Measurement & Control Research Department, National Engineering Laboratory of Petroleum Drilling Technology, Xi’an Shiyou University, Xi’an 710065, China; 2 State Key Laboratory of Petroleum Resources and Prospecting, China University of Petroleum, Beijing 102249, China

**Keywords:** fault diagnosis, embedded technology, acoustic logging, transducer array, data-driven technique, fault tree analysis

## Abstract

To improve the performance of acoustic logging tool in detecting three-dimensional formation, larger and more complicated transducer arrays have been used, which will greatly increase the difficulty of fault diagnosis during tool assembly and maintenance. As a result, traditional passive diagnostic methods become inefficient, and very skilled assemblers and maintainers are required. In this study, fault-diagnosis requirement for the acoustic logging tool at different levels has been analyzed from the perspective of the tool designer. An intelligent fault-diagnosis system consisting of a master-slave hardware architecture and a systemic diagnosis strategy was developed. The hardware system is based on the embedded technology, while the diagnosis strategy is built upon fault-tree analysis and data-driven methods. Diagnostic practice shows that this intelligent system can achieve four levels of fault diagnosis for the acoustic logging tool: System, subsystem, circuit board, and component. This study provided a more rigorous and professional fault diagnosis during tool assembly and maintenance. It is expected that this proposed method would be of great help in achieving cost reduction and improving work efficiency.

## 1. Introduction

**Figure 1:**
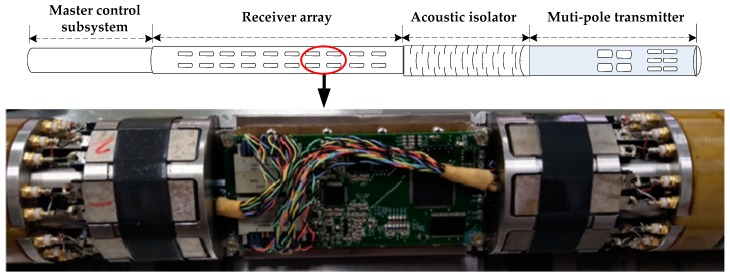
Structure of the 3-D acoustic logging tool.

In order to serve the oil and gas exploration industry better, most of the new acoustic logging tools have been equipped with multi-pole transmitter transducers and azimuthal receiving transducer arrays to achieve a high-precision detection of wider three-dimensional (3-D) formation [1,2,3]. Figure 1 shows the structure of a 3-D acoustic logging tool. This tool consists of four subsystems: A master control subsystem, a receiver array, an acoustic isolator, and a multi-pole transmitter. There are 80 acquisition channels in the 10 stations of the receiver array. Each station consists of eight receiving transducers, azimuthally placed at 45° intervals, and their related processing circuits [4]. Every two stations share the same receiving control node.

Fault diagnosis is indeed necessary to ensure the quality of all manufacturing processes and conduct efficient maintenance for the acoustic logging tool. Part-to-whole check during assembly and whole-to-part diagnosis during maintenance should be performed for this tool. However, along with the electromechanical integration design, large and complicated transducer arrays greatly increase the difficulty of fault diagnosis [5]. At the same time, assemblers and maintainers are often too unfamiliar with the tool’s internal structure to perform a fault diagnosis at a deeper level or in more stages without the help of special equipment. Therefore, designing a dedicated fault-diagnosis system for the assembler and maintainer from the perspective of the tool developer becomes very important. This will help them achieve a quick fault location and improve work efficiency.

Scholars have formulated a variety of qualitative and quantitative fault-diagnosis methods, based on a deep understanding of the diagnosed object’s characteristics. Fault tree analysis (FTA) is a graphical deductive tool that links given fault and its related lower-level events via a “top–down” approach. This method fully takes the system structure and its working principle into consideration and uses the minimum cut-set algorithm to evaluate the failures qualitatively and quantitatively [6]. It has been widely used in the reliability evaluation and fault diagnosis of hydraulic systems, power transformers, fuel cells, telecommunication devices, oil pipelines, and in other fields [7,8,9]. Data-driven fault diagnosis and prediction techniques have been developed rapidly, promoted by new technologies such as data mining and artificial intelligence [10,11,12]. According to differences in data source and processing, Dai et al. classified all data-driven fault-diagnosis methods into three categories: Model-based online-data-driven methods, signal-based methods, and knowledge-based historical-data-driven methods [13]. Moosavi et al. realized an automatic fault diagnosis and isolation method for the inclinometer system to improve its reliability through an additional skewed accelerometer [14]. Temer et al. designed an intelligent diagnostic method built on the Internet of things (IoT) and machine-learning technology for the predictive maintenance of downhole tools to avoid over-maintenance resulting from the traditional periodic approach [15]. At the same time, researchers have made efforts to improve the effectiveness of fault diagnosis and prediction by combining different parameters at different stages of specific systems, using genetic algorithms, neural networks, support vector machines, information fusion, and other similar techniques [16,17,18].

In terms of diagnosis for acoustic logging tools, a great deal of work has been accomplished. Several giant companies, such as Schlumberger and Baker Hughes, do not sell their logging tools and instead offer related field services. They build specialized teams to perform tool assembly, field service, and maintenance. However, this one-stop service is obviously not suitable for small research teams, nor good for oil companies aiming at cost reduction. Zhang et al. suggested building an expert system for tool fault diagnosis to cope with the lack of professional field maintainers [19]. They pointed out the difficulties in continuous updating and maintaining this expert system, which are needed to guarantee its effectiveness. Sun et al. designed a simulating source and tool bus interface to check multi-channel acquisition circuits and logging tools easily [20,21]. However, these solutions can perform only a partial diagnosis, and have poor comprehensiveness and hierarchy. Tu et al. tried to enhance the efficiency of fault-diagnosis systems for logging tools by utilizing an ART270 processor and Windows Embedded Compact (Windows CE) [22]. They simulated the tool’s working environment to examine its cable communication and field-bus node. However, such a system has limited diagnostic capability and is inconvenient to expand and upgrade. Ju et al. proposed a master-slave debugging system, which is based on Ethernet and embedded technology, to conduct fast and convenient diagnosis for different parts of the logging tool [5,23,24,25]. Their method possesses the advantages of high modularity and strong scalability. However, the matched diagnosis strategy, especially in terms of intelligence and adaptability, needs to be further improved.

On the basis of previous studies, the fault-diagnosis requirement of the acoustic logging tool was summarized, and an intelligent fault diagnosis system was designed in this paper from the perspective of the tool developer. In terms of hardware, this system was improved and upgraded based on the embedded master-slave architecture. In terms of software, multi-technology fusion, which combines fault-tree analysis, data-driven technique, and other methods, was applied for an intelligent diagnosis strategy. This system has achieved four levels of fault diagnosis for the tool: System, subsystem, circuit board, and component. Practice has shown that this special intelligent fault-diagnosis system by the tool developer could help workers achieve rigorous and professional fault diagnosis during assembly and maintenance, and improve the work efficiency greatly.

## 2. Design of the Intelligent Fault-Diagnosis System

### 2.1. Requirement Analysis of Fault Diagnosis

**Figure 2:**
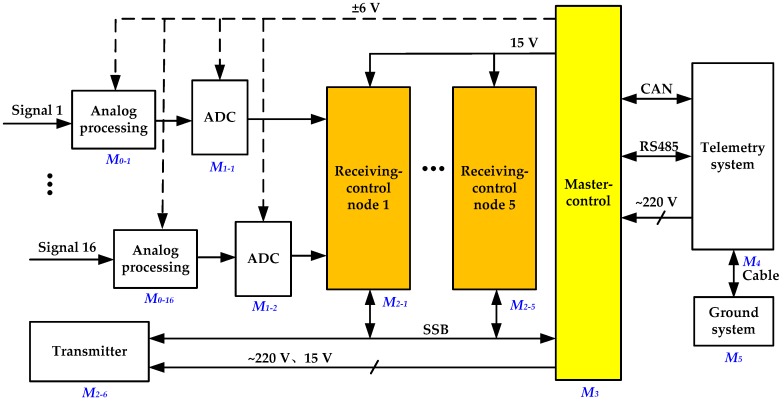
Electrical connections among all subsystems, and route of logging data.

Analyzing the structure and working principles of the tool and determining its diagnosis requirement are the foundations of designing an intelligent diagnosis system. As a further illustration of Figure 1, Figure 2 shows the detailed electrical connections among all subsystems, and the route of data in the acoustic logging tool. Managed by a receiving control node, 16 analog signals from the receiving transducers are first processed by programmable amplification and filtering, then converted to digital data, and assigned into a first input, first output (FIFO) memory of this node. The master control subsystem communicates with the transmitter and five receiving control nodes through a synchronous serial bus (SSB) [26]. All data from the 80 acquisition channels cached in those FIFOs are read and transmitted to the telemetry system by a dual-bus composed of controller area network (CAN) and RS485. Finally, the telemetry system delivers the encoded data to the ground system for subsequent operation. In addition, the power management module in the master control subsystem converts 220 V AC into low-voltage DC power supply of ±6 V and 15 V for corresponding modules in the transmitter and receiver.

This structure requires a diagnosis at four levels, i.e., component, circuit board, subsystem, and system, in part-to-whole order in the process of tool assembly and in the opposite order during maintenance. Details of the diagnosis at the four levels are as follows:

1. System diagnosis: Diagnosing the whole downhole tool;
2. Subsystem diagnosis: Checking the master control subsystem (*M_3_*), transmitter (*M_2-6_*), and receiver nodes (*M_2-1_* to *M_2-5_*);
3. Circuit-board diagnosis: Testing the critical, complex, or batch-used circuit boards in the subsystems, such as the master control board, analog-processing board, and transmitting control board;
4. Component diagnosis: Performing a strict examination of the characteristics of some special components, such as the consistency of the transducers and the high temperature stability of flash memories.

### 2.2. Principles of Intelligent Fault Diagnosis

When fault occurs in a module of the tool, abnormal data, which are different from the data under normal conditions, are presented at the terminal (*M_5_*). This principle is the foundation of fault diagnosis, which is based on fault-tree analysis and data-driven methods.

Design principles of this intelligent fault-diagnosis system can be described as follows: (1) Regarding the hardware: The designed functional modules need to be attached to the embedded master-slave architecture and combined into diagnosis interfaces at different levels. Instead of corresponding subsystems or modules, these interfaces provide the inputs that are necessary for the diagnosed object to work normally and check its outputs. (2) Regarding the diagnosis strategy: According to the tool structure and its working protocol, different diagnosis modes and their simulation data are designed in each of the modules of the tool. By processing data at the terminal through methods like time-frequency analysis and normalization, the fault source can be found step by step using the fault-tree idea, and thus the intelligent fault diagnosis based on multi-technology fusion can be achieved.

**Figure 3:**
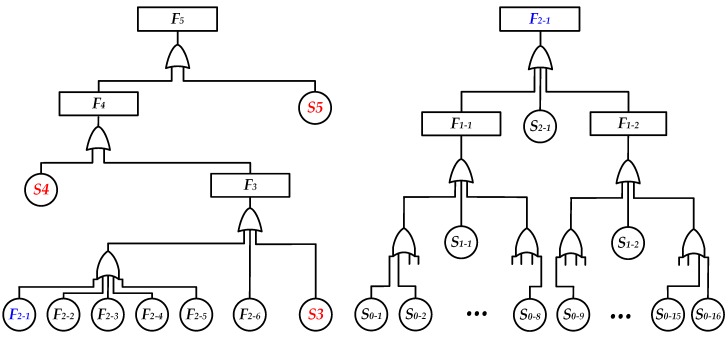
Fault-tree diagram of the tool (part).

Figure 3 shows the tool’s fault-tree diagram on the basis of the tool structure and the route of logging data. The detailed steps of fault-tree construction for the diagnosis strategy are as follows:

1. The tool system can be divided into several modules, which are located at different positions and levels in the tool, and have certain independences and relationships with each other, such as *M_0_* to *M_5_* in Figure 2.
2. The relative upstream and downstream modules are defined according to the specific path sequence of logging data when the tool is in operation. For example, *M_3_* is the downstream module of *M_2_* and is the upstream module of *M_4_*. Failures from the upstream modules will propagate to the downstream modules. If any module fails, data in the ground terminal (*M_5_*) will be abnormally displayed.
3. A Boolean variable *S_i_* is used to represent the failure condition of module *M_i_*, and a Boolean variable *F_i_* to represent the failure condition of output *M_i_*. Then, the relationships between *S_i_* and *F_i_* can be described by the fault-tree diagram shown in Figure 3. Their mathematical relationships are shown in Equations (1) and (2), where *F_i_* and *S_i_* are equal to logic 1 when a fault occurs.

(1) $$F_{i}=S_{i}\cup F_{i-1},\;i=1,2,3,4,5,$$

(2) $$F_{0}=S_{0}.$$

It can be seen from Figure 3 that failures caused by the ground system (*M_5_*), telemetry system (*M_4_*), and master control subsystem (*M_3_*) are global faults, while failures from each of the analog processing channels in *M_0_*, analog-to-digital converters (ADC) in *M_1_*, and receiving control nodes in *M_2_* are local faults at different levels. Therefore, in order to achieve an intelligent fault diagnosis, it is very effective to design different diagnosis modes and their simulation data in each of the modules to replace the outputs of the upstream modules (make *F_i-1_* = 0). By doing so, a fault can be gradually located in the downstream and upstream modules according to the value of *F_i_*. If *F_i-1_* = 0 and *F_i_* = 0, it means that failure occurs in the upstream modules; otherwise, the failure occurs in the *M_i_* module.

### 2.3. Hardware Design of the Fault-Diagnosis System

In order to achieve an intelligent diagnosis, an embedded front-end machine based on the ARM7 microprocessor and μClinux system was constructed in this study. According to the diagnosis requirement, functional modules, such as the power management module and the analog signal generator, were designed and combined into diagnosis interfaces for the acoustic logging tool at four levels: System, subsystem, circuit board, and component. By replacing the specific subsystems or functions of different modules in the tool, these interfaces could realize an effective fault diagnosis during tool assembly and maintenance.

#### 2.3.1. Overall Design of the Hardware System

**Figure 4:**
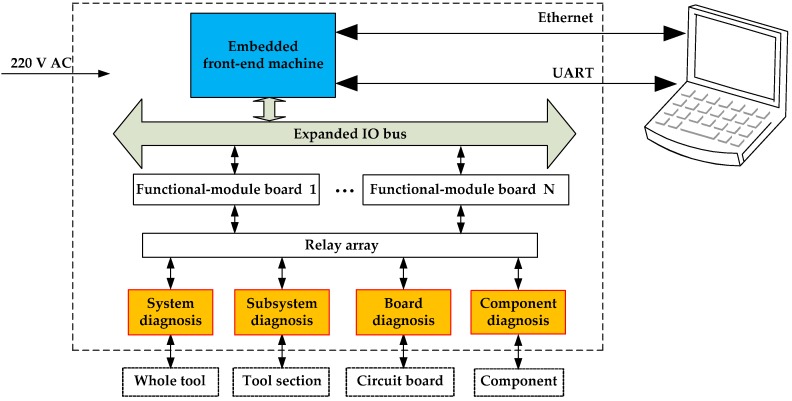
Overall design of the hardware system.

**Figure 5:**
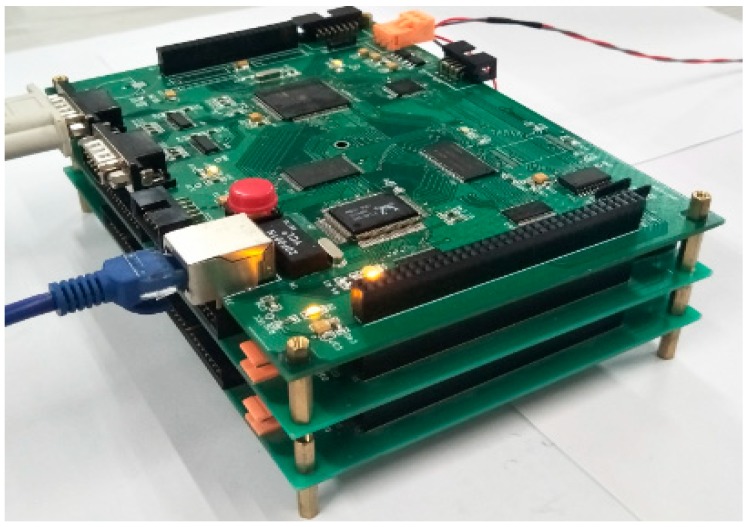
Structure of building-block stacking for the system.

Figure 4 describes the overall design of the hardware system. This system consists of a host computer, an embedded front-end machine, functional-module boards, a relay array, and other components. The host computer software achieves several functions such as network communication and data drawing. Using a S3C44B0X microprocessor for control purpose, the embedded front-end machine is the core of the whole system [27]. It communicates with the host computer through Ethernet and serial ports [28], and accesses the functional-module boards via an extended IO bus. The extended IO bus is actually an ARM external read–write bus that presents a pinhole structure similar to that of a PC104 bus [29]. Utilizing this bus, circuit boards of the functional modules, along with the embedded front-end machine, form a structure of building-block stacking in space, as shown in Figure 5. This not only makes the system more compact, but also facilitates the distributed control. Different functional boards implement their functions such as analog signal generation, signal acquisition, bus interface, and power management. By combining these functional boards according to the requirement, diagnosis interfaces for different purposes at four levels can be designed for the acoustic logging tool. The application of relay array enables this system to support hot swap and signal isolation. During testing, the diagnostic object (e.g., the whole tool, a subsystem, and a circuit board) should be connected to the corresponding diagnosis interface through an adapter to perform a rapid fault diagnosis.

#### 2.3.2. Design of Functional Modules

According to the hardware requirement of the diagnosis system, the EP2C20Q240C8 field-programmable gate array (FPGA) was chosen as the control core of all functional modules [30]. These modules, such as the multi-bus communication, analog signal generator, multi-channel signal acquisition, power management, transducer excitation, and flash memory testing, were designed to implement specific functions.

**Figure 6:**
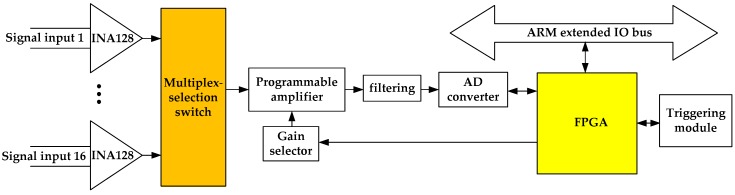
Schematic diagram of the multi-channel signal acquisition module.

In order to illustrate the design methods of functional modules, we took the multi-channel signal acquisition module as an example. Figure 6 shows the schematic diagram of this module. It has a matched submodule in FPGA to connect with the extended IO bus, and can provide differential signal acquisition for 16 channels. Based on a precise and low-power instrumentation amplifier (INA128), the pre-processing circuit aims to achieve the following amplification for the acquired signal. The multiplex-selection switch selects one signal from all signals to be output to the next-stage processing module under the control of FPGA. The programmable amplifier realizes automatic gain amplification for the signals by selecting a matching feedback resistor. After filtering, the amplified analog signal is converted into digital signal and then comes into the FPGA. In addition, this module has a triggering function to synchronize with other modules for coordinated operation.

#### 2.3.3. Diagnosis Interfaces at Different Levels

According to the principle of providing inputs to the diagnosed object and testing its outputs, diagnosis interfaces can be combined using different functional modules based on their requirements. These interfaces can be divided into four levels: System, subsystem, circuit board, and component. The system-diagnosis interface is meant for the debugging of the whole tool. Three subsystem-diagnosis interfaces were designed for the master control subsystem, transmitter, and receiver. The diagnosis interfaces at circuit-board level are mainly meant for the master control board, analog processing board, and transmitting control board. Finally, the component-diagnosis interfaces are meant for testing special components, such as transducers and flash memories.

**Figure 7:**
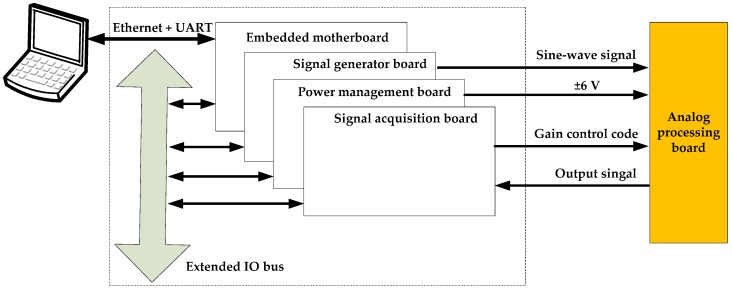
Diagnosis interface for the analog processing board.

The analog processing board in the acoustic logging tool is a critical, complex, and batch-used circuit board, therefore its diagnosis interface is taken as an example to show how the interfaces were designed in this research. By checking the passband characteristics and programmable amplification of each channel, availabilities and consistencies of all 40 analog processing boards used in the tool can be evaluated. As shown in Figure 7, the embedded motherboard and three functional boards are included in the diagnosis interface. The power management board generates and monitors the output of ±6 V to guarantee a reliable power supply for the checked board. According to the selection in the control software, the signal generator board can generate sine-wave signals of different frequencies and amplitudes as standard inputs for the diagnosis. Two operations need to be done by the signal acquisition board, i.e., providing different combinations of gain-control codes for the analog processing board, and acquiring its output signals and uploading them to the host computer. In addition, signals at different test points on the diagnosed board can be acquired for further fault location.

### 2.4. Software Design of the Fault-Diagnosis System

The software contains three parts to achieve an intelligent diagnosis. The first part consists of the control programs of all functional modules in FPGA based on the very high-speed integrated circuit (VHSIC) hardware description language (VHDL). The second part consists of programs in the front-end machine, including the transplant of the μClinux system, TCP/IP network communication based on socket mechanism, and an access driver for the extended IO bus [31]. The third and most important part is the intelligent diagnosis strategy, which uses fault-tree analysis and data-driven methods, on the host computer.

**Figure 8:**
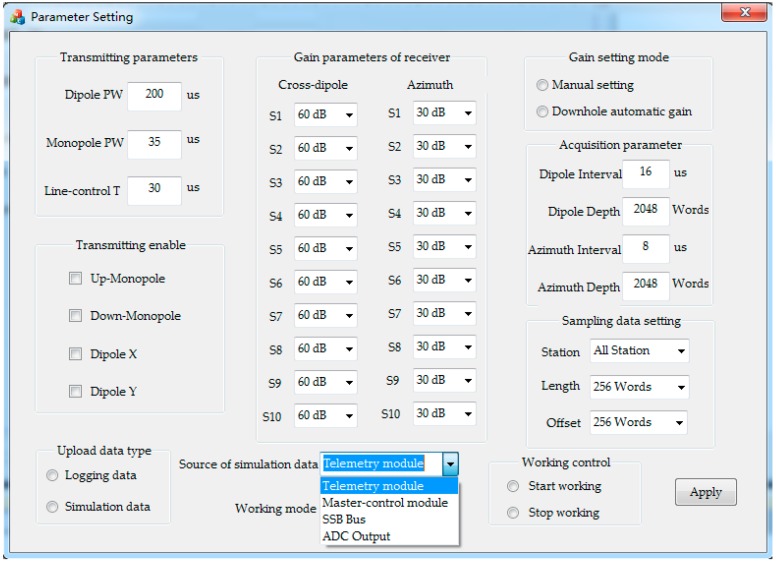
Parameter setting for intelligent diagnosis.

Figure 8 shows the parameter-setting interface designed for the diagnosis system according to the tool’s working protocol. Users can configure the following settings to achieve the isolated intelligent diagnosis of specific modules. (1) Set transmitter parameters, such as excitation pulse width of monopole and dipole transducers, and enable controls to select whether these transducers are allowed to work. (2) Set receiver parameters, such as sampling depth and interval, and programmable gains of 10 receiving stations in azimuth and cross-dipole modes. Among these, gains can range from 0 dB to 84 dB with a step of 6 dB in both downhole automatic and ground manual setting modes. (3) Choose data type and its settings for selective uploading, for example, select to upload logging data or simulation data, and select the stations, start position, and length of data uploading. (4) Select the source of simulation data in the tool, such as telemetry module, SSB bus, master-control module, and ADC output.

**Figure 9:**
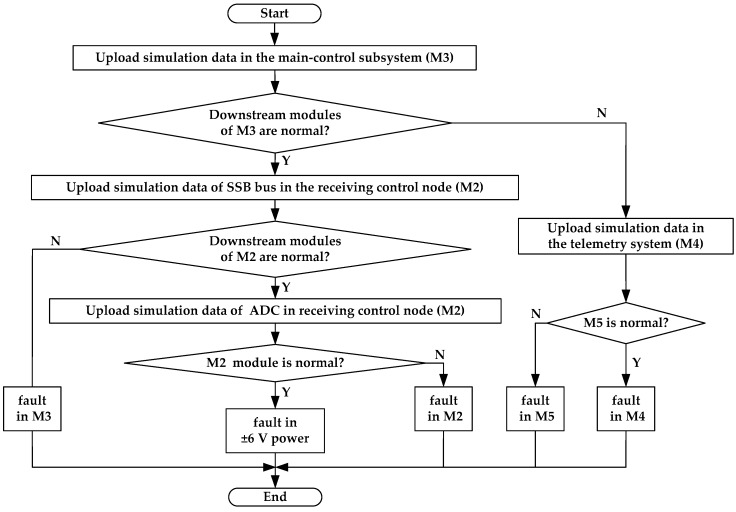
Flowchart of the intelligent diagnosis for global error in acquired data.

Figure 9 shows the flowchart of the intelligent diagnosis by this system when a global error occurs in the acquired data. According to the flow path of the acquired data, the diagnosis strategy is designed using fault-tree analysis and data-driven methods. For a convenient diagnosis, many diagnosis modes and their simulation data have been added to different modules when this tool was being developed. When the intelligent diagnosis is started, simulation data from the master control subsystem (*M_3_*), telemetry system (*M_4_*), SSB bus, and ADC modules are uploaded selectively, allowing the fault location to be confirmed gradually.

## 3. Intelligent Diagnosis Results

The intelligent system can achieve four levels of fault diagnosis for the acoustic logging tool, as shown in Table 1. In this paper, debugging for the analog processing board and fault diagnosis for the global error in acquired data were taken as examples to illustrate the intelligent fault diagnosis during tool assembly and maintenance.

### 3.1. Debugging for the Analog Processing Board

The analog processing board can be checked from two aspects to confirm the fault location and evaluate the consistency of the processing channels with other boards, using the matched diagnosis interface shown in Figure 7. One aspect was measuring the passband characteristics of the processing channels under the same gain-control condition. The other was testing the gains of the processing channels under different gain-control conditions and checking their matching.

**Figure 10:**
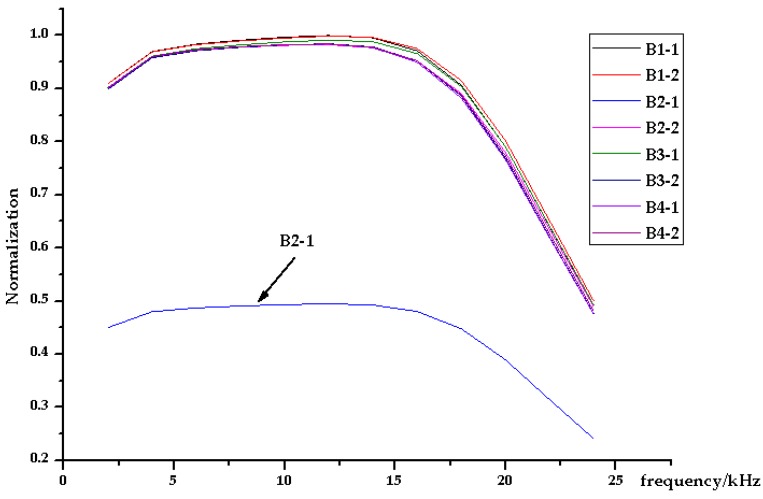
Normalized passband characteristics of the analog processing channels.

Figure 10 shows the normalized passband characteristics of eight channels on four boards in the same receiving station after frequency-sweeping testing. The passband characteristics of the B2-1 channel was seen to be different from those of the other seven channels, with signals having amplitudes that were almost half of the expected values in the range of 2 kHz to 18 kHz. Table 2 compares gain-control codes, expected gains, and actual gains of the eight channels at 10 kHz. Testing results showed that, under all gain-control codes, the B2-1 channel had abnormal actual gains, which were inconsistent with those of the other seven channels. The result of the diagnosis indicates that the fault occurred in the pre-processing module of the B2-1 channel. It was found that the 100 Ω feedback resistance was falsely welded to 49.9 Ω in this module. The passband characteristics and gains of the B2-1 channel returned to normal after the resistance was replaced.

### 3.2. Intelligent Diagnosis for Abnormality in the Acquired Data

**Figure 11:**
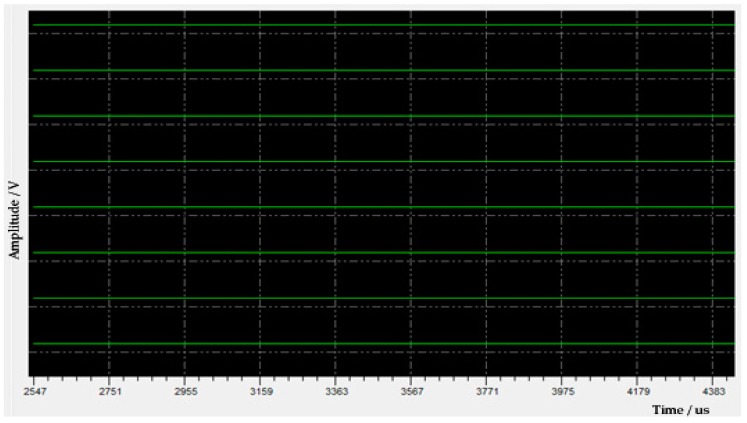
Abnormal environmental noise data uploaded by the tool.

**Figure 12:**
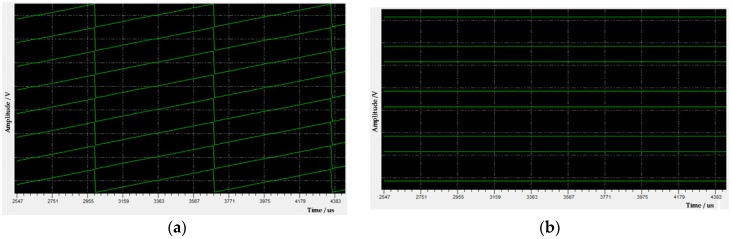

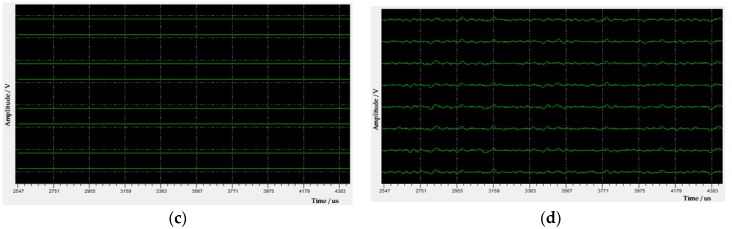
Waveforms of the uploaded data during diagnosis: (**a**) Simulation data from the M3 module; (**b**) simulation data from the synchronous serial bus (SSB) bus; (**c**) simulation data from analog-to-digital converters (ADC) output; and (**d**) normal noise waveforms.

(1) Fault features. During a pre-logging checking in the workshop, the environmental noise data uploaded by the acoustic logging tool were found to be abnormal. The fault features can be summarized as follows. (a) All eight waveforms in each receiving station were positive straight lines, as shown in Figure 11, which did not match the expected noise waveforms. (b) These waveforms did not change when the gain setting mode was modified. (c) When power-on resets again, the fault did not disappear. On the surface, this fault seemed to be global, and might be caused by the failure of the receiver (*M2*) or of its downstream modules. However, further diagnosis is needed to confirm the specific fault location and its cause.

(2) Intelligent fault diagnosis. The system performed an automatic diagnosis according to the intelligent diagnosis strategy for the abnormality of acquired data in Figure 9. Figure 12 displays the waveforms of the uploaded data on the host computer during diagnosis. Sawtooth waveforms in Figure 12a were correct simulation data from the master control subsystem (*M_3_*). They indicate that the downstream modules of *M_3_* were normal. Figure 12b shows the simulation data for the SSB bus testing in receiving control node (*M_2_*). Waveforms of the 1st, 3rd, 5th, and 7th channels in each station were positive straight lines, while the waveforms of the 2nd, 4th, 6th, and 8th channels were negative straight lines. These observations were consistent with the expectations, indicating that the SSB bus between *M_3_* and *M_2_* working normally. The uploaded ADC simulation data generated by the receiving control node (*M_2_*) can be seen in Figure 12c. Waveforms of all channels were opposite to those of the SSB bus in Figure 12b. This means that the control module of ADC was working well. These expected waveforms in Figure 12a–c proved that the fault occurred either in the ADC module (*M_1_*) or in its upstream module (*M_0_*). Since this abnormality was global, and the submodules in *M_0_* and *M_1_* had a certain degree of independence, it was suspected that the ±6 V power module for *M_0_* and *M_1_* in the master control subsystem had failed. A load test was performed on the ±6 V power module using the power diagnosis interface. It was found that the load capacity of +6 V was insufficient. When a 5 Ω resistive load was applied, the +6 V power module was only producing 2.17 V, while the input voltage of this power module was normally 16.14 V. After the power module LH42094 was replaced, the load capacity of +6 V became normal, and the whole tool was enabled to work normally and uploaded the expected noise waveforms, as shown in Figure 12d.

## 4. Discussion

Checking the batch-used analog processing board is a time-consuming and laborious task. The global abnormality of the acquisition data on the surface was finally traced back to the damage of the power module in the master control subsystem. When the fault-diagnosis system based on embedded technology and intelligent diagnosis strategy was used, a quick and accurate fault location was achieved in the examples provided in this work. Such performance cannot be achieved by a traditional instrument-based passive diagnosis method. Therefore, designing an effective fault diagnosis system by the tool developer was significant. It was demonstrated that the combination of the qualitative and quantitative diagnosis methods, such as the fault tree analysis and data-driven technique, could make the fault-diagnosis system more rigorous and effective during tool assembly and maintenance.

## 5. Conclusions

The use of large and complex transducer arrays and the electromechanical integration design in acoustic logging tools made fault diagnosis more difficult and time consuming. The industrialization of this tool requires specialized fault-diagnosis equipment. Therefore, it is necessary to design such an equipment to help tool assemblers and maintainers determine the fault location quickly and accurately. This is helpful in improving the overall work efficiency and reducing costs.

From the perspective of the tool developer, the study on the intelligent fault-diagnosis system was done as follows. (1) Fault-diagnosis requirements at different levels and stages for the acoustic logging tool were summarized. (2) A hardware platform was developed and upgraded based on the master–slave embedded architecture technology. (3) An intelligent diagnosis strategy was designed by combining the fault-tree analysis and data-driven methods. This system achieved an intelligent diagnosis at four levels of the tool: System, subsystem, circuit board, and component. This system could significantly improve the hierarchy, rigorousness, on-site operation, and specialization of fault diagnosis during tool assembly and maintenance.

In short, only by accounting for the requirement of an efficient fault-diagnosis during tool development, by improving the intelligent diagnosis system based on multi-technology fusion continuously, by conducting fault diagnosis throughout the tool lifecycle, and by developing it towards predictive intelligent diagnosis based on condition monitoring, we could further improve work efficiency and quality, and promote the development, manufacture, application, and maintenance of acoustic logging tools.

## Figures and Tables

**Table 1:** Diagnosis items at four levels for the tool.

Diagnosis Level		Diagnosis Items
System	Abnormality of the tool to generate and receive acoustic signal and transmit data according to the protocol	
Subsystem	Master control Subsystem	(1) Abnormality of the communication with the telemetry system, receiver, and transmitter; (2) output abnormality of the low-voltage DC power supply (+6 V, 15 V)
	Receiver	Abnormality of 80 acquisition channels and their consistencies
	Transmitter	Excitation abnormality of the muti-pole transducers
Circuit board	Master control board	Abnormality of the interfaces of CAN, RS485, SSB
	Analog processing board	Abnormality of passband characteristics and gain control
	Transmitting control board	Consistency abnormality of SSB command and transmitting control logic combination
	Other boards, such as the acquisition control board and power supply board	
Component	Performance abnormality of pulse transformers, consistency of transmitting/receiving transducers, stability of memory, etc.	

**Table 2:** Gain table of the analog processing channels.

Gain Code	00	01	02	03	04	05	06	07	10	20	30
Expected gain	1.00	2.00	4.00	8.00	16.00	32.00	64.00	128.00	64.00	2.08	128.00
B1-1 gain	1.04	2.08	4.13	8.50	17.08	34.15	68.38	135.48	69.54	2.08	139.20
B1-2 gain	1.04	2.08	4.12	8.53	17.09	34.27	68.46	136.09	69.89	2.06	139.24
B2-1 gain	0.53	1.03	2.05	4.25	8.46	17.06	34.07	67.98	34.78	1.03	69.45
B2-2 gain	1.03	2.05	4.06	8.44	16.92	33.92	67.75	134.91	68.93	2.07	137.73
B3-1 gain	1.03	2.06	4.09	8.50	17.03	34.19	68.30	135.77	69.65	2.06	138.62
B3-2 gain	1.03	2.05	4.07	8.45	16.96	34.00	67.99	134.68	68.73	2.05	137.50
B4-1 gain	1.03	2.05	4.07	8.45	16.95	34.01	67.91	133.50	66.75	2.05	137.80
B4-2 gain	1.03	2.05	4.07	8.46	16.94	34.00	67.91	134.41	68.57	2.06	137.92
